# Comparison of trends of inpatient charges among primary and revision shoulder arthroplasty over a decade: a regional database study

**DOI:** 10.1016/j.jseint.2023.08.001

**Published:** 2023-09-11

**Authors:** Trevor Simcox, Aidan G. Papalia, Brandon Passano, Utkarsh Anil, Charles Lin, William Mitchell, Joseph D. Zuckerman, Mandeep S. Virk

**Affiliations:** b Department of Orthopedic Surgery, NYU Langone Health, New York, NY, USA; a Department of Orthopedic Surgery, NYU Langone Hospital - Long Island, Mineola, NY, USA

**Keywords:** Shoulder arthroplasty, Reverse total shoulder arthroplasty, Anatomic total shoulder arthroplasty, Hemiarthroplasty, Trends, Inpatient charges, Utilization, Cost effectiveness

## Abstract

**Background:**

This study examined trends in inpatient charges for primary anatomic total shoulder arthroplasty (aTSA) and reverse total shoulder arthroplasty (rTSA), hemiarthroplasty (HA), and revision total shoulder arthroplasty (revTSA) over the past decade.

**Methods:**

The New York Statewide Planning and Research Cooperative System was queried for patients undergoing primary aTSA, rTSA, HA, and revTSA from 2010 to 2020 using International Classification of Diseases procedure codes. The primary outcome measured was total charges per encounter. Secondary outcomes included accommodation and ancillary charges, charges covered by insurance, and facility volume. Ancillary charges were defined as fees for diagnostic and therapeutic services and accommodation charges were defined as fees associated with room and board. Subgroup analysis was performed to assess differences between high- and low-volume centers.

**Results:**

During the study period, 46,044 shoulder arthroplasty cases were performed: 18,653 aTSA, 4002 HA, 19,253 rTSA, and 4136 revTSA. An exponential increase in rTSA (2428%) and considerable decrease in HA (83.9%) volumes were observed during this period. Total charges were the highest for rTSA and revTSA and the lowest for aTSA. Subgroup analysis of revTSA by indication revealed that total charges were the highest for periprosthetic fractures. For aTSA, rTSA, and HA, high-volume centers achieved significantly lower total charges compared to low-volume centers. Over the study period, total inpatient charges increased by 57.2%, 38.4%, 102.4%, and 68.4% for aTSA, rTSA, HA, and revTSA, outpacing the inflation rate of 18.7%.

**Conclusion:**

Total inpatient charges for all arthroplasty types increased dramatically from 2010 to 2020, outpacing inflation rates, but high-volume centers demonstrated greater success at mitigating charge increases compared to low-volume centers.

The United States spends significantly more on healthcare than other developed nations, with a national healthcare expenditure of $4.1 trillion.^6^ In response, US policymakers are pressuring health-care institutions to develop cost-mitigating strategies. While lower extremity arthroplasty accounts for the largest healthcare expenditure of any Medicare diagnosis group, there is a need for cost-mitigation strategies for total shoulder arthroplasty (TSA).^1^^,^^10^^,^^13^^,^^29^ From 2011 to 2017, the incidence of primary TSA rose by 103.7% and is projected to reach an annual volume of 170,000-350,000 by 2025.^29^ Revision TSA (revTSA) surgical volume has paralleled that of primary TSA, resulting in a considerable economic burden to the United States, costing $205 million in 2017.^10^^,^^13^ To address this, policymakers have insisted on the development of cost-mitigation strategies for shoulder arthroplasty.^16^

Prior studies have demonstrated a direct correlation between surgical volume and value in TSA.^15^^,^^24^ Surgical centers performing higher procedure volumes are associated with lower charges, hospitalization length, complication rate, and readmission rate.^24^ Previous economic analyses have primarily focused on Medicare charges in national databases, but there exists a paucity of studies comparing patient-level charges at the state-level.^14^ Patient-level charges better represent resource utilization as opposed to indirect top-down approaches, providing more informative charge comparisons. Some state level databases provide a comprehensive collection of patient-level records, regardless of insurance coverage.

The purpose of this study was to compare inpatient charges for primary anatomic TSA (aTSA), reverse TSA (rTSA), and hemiarthroplasty (HA) and revTSA and review trends in New York’s Statewide Planning and Research Cooperative System (SPARCS) database over the last decade. We hypothesized that rTSA and revTSA would demonstrate the highest inpatient charges, and centers with higher procedural volumes would be associated with lower patient-level charges.

## Materials and methods

### Database

The SPARCS database is a comprehensive data reporting system for the Department of Health that compiles all inpatient and outpatient preadjudicated claims within New York State. The database provides patient-level information related to demographics, admission diagnoses, inpatient and outpatient treatments, and hospital charges.^19^ SPARCS has previously been used to study total joint arthroplasty as it pertains to hospital charges and trends across the state of New York.^11^ The results of prior studies that utilized SPARCS paralleled those that utilized Medicare provider utilization and payment data.^31^ SPARCS contain patient-identifiable demographic data, and institutional review board approval was obtained.

### Generation of study groups

Patients undergoing primary aTSA, rTSA, HA, and revTSA between the years 2010 and 2020 were identified using the Current Procedural Terminology and International Classification of Diseases, Ninth (ICD-9) and Tenth (ICD-10) Revision procedure codes. ICD-9 and ICD-10 diagnosis codes were used to identify the surgical indication. We excluded patients with oncologic diagnoses. Patients were assigned to one of four of the following cohorts: aTSA, rTSA, HA, and revTSA. Subgroup analyses within the revTSA cohort were performed based on diagnosis. For each of the four cohorts, subgroup analysis was also performed comparing charges at high volume to those at low volume centers. The case threshold for a high-volume center was defined as greater than 554 cases over the study period, coinciding with the top 90th percentile in surgical volume.

### Variables studied

Patient demographics, diagnosis codes, procedure volumes, and charges were collected. The primary outcome measure was total charges for each hospital encounter. Hospital charges were measured in current US dollars (USD), representing charges in dollars unadjusted for inflation. Secondary outcome measures included the breakdown of total charges into ancillary and accommodation charges, charges covered by insurance, noncovered charges, year of procedure, and facility shoulder arthroplasty volume. Both ancillary and accommodation charge subcategories were included to provide a more detailed understanding of charges associated with each encounter. The Center for Medicare & Medicaid Services defines ancillary service charges as those pertaining to laboratory, radiology, pharmacy, perioperative, operative, therapy, implant, and special item services (Table I).^7^^,^^18^ Accommodation charges were defined as fees associated with patient room and board (Table I).

**Table I:** Components of ancillary and accommodation charges.

Ancillary charges	Accommodation charges
Physician fees (surgeon, anesthesiologist, hospitalist, etc.)	Room and board
Hospital anesthesia services	Private & semi-private rooms
Intraoperative services	Intensive care unit
Operating room staff wages	Nursing & staff wages
Operating room equipment	Laundry services
Disposable surgical supplies	Stock items & medical supplies
Medications	Dressings & wound care supplies
Postanesthesia & postoperative recovery	Sequential compression devices
Special items	Personal hygiene products
Implants	Indirect hospital costs
High-cost surgical dressings	Administrative costs
Orthotics	Waste management
Other implanted surgical material (sutures, etc.)	Heat, ventilation, and air conditioning
Laboratory & pathology services	Other overhead expenses
Pharmacy services	
Radiology services	

### Statistical analysis

Statistical analysis was performed using *R* statistical software (R version 4.2.0; Vienna, Austria). Multivariate analysis was performed to compare total charges, ancillary charges, accommodation charges, charges covered by insurance, and shoulder arthroplasty volumes between cohorts. Demographic differences between groups were also compared. Subgroup analysis was performed to assess differences between high-volume and low-volume centers. Statistical differences between groups were identified using the Kriskal-Wallis rank sum, Pearson’s chi-squared, or Welch two-sample t-test. *P* values <.05 were considered statistically significant, and Bonferroni corrections were performed where appropriate.^23^^,^^25^

## Results

A total of 46,044 shoulder arthroplasty procedures were performed in New York State during the study period, of which 18,653 patients underwent primary aTSA, 19,253 underwent primary rTSA, 4002 underwent primary HA, and 4136 underwent revTSA (Table II).

**Table II:** Demographics.

	Overall	Anatomic TSA	Hemiarthroplasty	Reverse TSA	Revision TSA	*P* value
	*n = 46,044*	*n = 18,653*	*n = 4002*	*n = 19,253*	*n = 4136*	
Age (yr)	68.67 ± 10.29	66.47 ± 9.63	66.36 ± 14.24	71.91 ± 8.81	65.74 ± 10.96	**<.001**∗
Gender						
Female	26,328 (57%)	9328 (50%)	2500 (62%)	12,320 (64%)	2180 (53%)	**<.001**†
Male	19,716 (43%)	9325 (50%)	1502 (38%)	6933 (36%)	1956 (47%)	
Race						**<.001**†
White	38,732 (84%)	15,808 (85%)	3179 (79%)	16,299 (85%)	3446 (83%)	
Other or unknown	2389 (5.2%)	983 (5.3%)	250 (6.2%)	947 (4.9%)	209 (5.1%)	
Black	2319 (5.0%)	935 (5.0%)	256 (6.4%)	890 (4.6%)	238 (5.8%)	
Hispanic	2166 (4.7%)	797 (4.3%)	247 (6.2%)	916 (4.8%)	206 (5.0%)	
Asian	328 (0.7%)	87 (0.5%)	63 (1.6%)	154 (0.8%)	24 (0.6%)	
Native American	110 (0.2%)	43 (0.2%)	7 (0.2%)	47 (0.2%)	13 (0.3%)	
Insurance						**<.001**†
Medicare	26,991 (59%)	9656 (52%)	2168 (54%)	12,992 (67%)	2175 (53%)	
Private health insurance	15,112 (33%)	7563 (41%)	1390 (35%)	4745 (25%)	1414 (34%)	
Worker's compensation	2360 (5.1%)	845 (4.5%)	199 (5.0%)	978 (5.1%)	338 (8.2%)	
Medicaid	1083 (2.4%)	383 (2.1%)	177 (4.4%)	365 (1.9%)	158 (3.8%)	
Other	498 (1.1%)	206 (1.1%)	68 (1.7%)	173 (0.9%)	51 (1.2%)	
Length of stay (d)	2.23 (2.73)	1.75 (1.29)	4.09 (5.60)	2.18 (2.42)	2.88 (3.64)	**<.001**∗
Discharge disposition						**<.001**†
Home	40,465 (88%)	17,499 (94%)	2963 (74%)	16,365 (85%)	3638 (88%)	
Skilled nursing facility	4620 (10%)	953 (5.1%)	831 (21%)	2429 (13%)	407 (9.8%)	
Inpatient rehab	751 (1.6%)	155 (0.8%)	167 (4.2%)	366 (1.9%)	63 (1.5%)	
Other	138 (0.3%)	34 (0.2%)	29 (0.7%)	53 (0.3%)	22 (0.5%)	
Transfer	70 (0.2%)	12 (<0.1%)	12 (0.3%)	40 (0.2%)	6 (0.1%)	
Indication for surgery						**<.001**†
Primary and secondary arthritis	30,973 (70%)	16,756 (91%)	1607 (45%)	11,754 (63%)	856 (23%)	
Trauma	4358 (9.8%)	178 (1.0%)	1446 (41%)	2538 (14%)	196 (5.3%)	
Rotator cuff arthropathy	2432 (5.5%)	104 (0.6%)	15 (0.4%)	2180 (12%)	133 (3.6%)	
Prosthetic complication	2207 (5.0%)	31 (0.2%)	28 (0.8%)	185 (1.0%)	1963 (53%)	
Other shoulder pathology	4306 (9.7%)	1325 (7.2%)	462 (13%)	1945 (10%)	574 (15%)	

*TSA*, total shoulder arthroplasty.

Bolded *P*-values denote a statistically significant value.

∗ Kruskal-Wallis rank sum test.

† Pearson chi-squared test.

There were demographic differences between groups (Table II). Patients undergoing revTSA were older (*P* < .001) and more likely to be female (*P* < .001). The rTSA cohort had a higher proportion of patients with Medicare insurance than that of the other cohorts (*P* < .001). Patients undergoing primary aTSA were most likely to be discharged home (*P* < .001). The HA cohort had the smallest proportion of white patients of any cohort (*P* < .001). The HA cohort had the longest length of stay at 4.09 days, compared to 1.75 days for aTSA, 2.18 for rTSA, and 2.88 for revTSA (*P* < .001). There were significant differences in surgical indication between groups (*P* < .001, Table II). A majority of patients undergoing aTSA were diagnosed with primary or secondary arthritis. In contrast, 45% of HAs were performed for arthritis, followed by 41% for trauma, and 13% for other shoulder pathology. For the rTSA cohort, 63% had arthritis, 14% had trauma, 12% had RTC arthropathy, 10% had other shoulder pathology, and 1% had complications of prior hardware. For revTSA, 53% underwent the procedure for a prosthetic complication, 23% for arthritis, 15% for other, 5.3% for trauma, and 3.6% for RTC tears.

Inpatient charges were then evaluated between cohorts (Table III). Total inpatient charges were highest for rTSA ($66,438), followed by revTSA ($64,766), HA ($55,315), and finally aTSA (50,165, *P* < .001). Ancillary charges were also highest for rTSA ($58,075), followed by revTSA ($52,332), aTSA ($43,402), and HA ($40,914). Accommodation charges were the highest for HA ($14,401), then revTSA ($12,434), rTSA ($8364), and aTSA ($6763). The HA cohort had the highest mean noncovered total ($411, *P* < .001), ancillary ($226, *P* < .001), and accommodation ($186, *P* < .001) charges of all the cohorts (Table III).

**Table III:** Inpatient charge analysis for primary and revision TSA.

	Anatomic TSA	Hemiarthroplasty	Reverse TSA	Revision TSA	*P* value∗
	*n = 18,653*	*n = 4002*	*n = 19,253*	*n = 4136*	
Accommodation total charges	6763.11 ± 6985.71	14,401.95 ± 28,672.99	8363.81 ± 13,801.11	12,433.54 ± 21,784.35	**<.001**
Noncovered accommodation charges	62.28 ± 569.21	185.52 ± 2661.95	45.57 ± 691.78	80.98 ± 1146.21	**<.001**
Ancillary total charges	43,402.12 ± 28,092.59	40,913.94 ± 37,901.34	58,074.75 ± 40,148.73	52,332.18 ± 39,300.21	**<.001**
Noncovered ancillary charges	63.11 ± 1738.80	225.65 ± 3702.04	54.78 ± 1851.42	86.88 ± 2350.53	**<.001**
Total charges	50,165.22 ± 30,744.20	55,315.89 ± 59,877.64	66,438.56 ± 46,436.97	64,765.72 ± 51,432.04	**<.001**
Total noncovered charges	125.39 ± 2136.12	411.17 ± 5828.68	100.35 ± 2228.23	167.86 ± 2921.25	**<.001**

*TSA*, total shoulder arthroplasty.

Bolded *P*-values denote a statistically significant value. All charges listed in US Dollars.

∗ Kruskal-Wallis rank sum test.

Charges were then evaluated within the revTSA cohort based on surgical indication (Table IV). Within the revTSA cohort, 359 patients were indicated for aseptic loosening, 1024 for implant failure, 627 for instability, 103 for periprosthetic fracture, 640 for periprosthetic joint infection (PJI), and 240 for rotator cuff (RTC) deficiency or rupture. Total charges were highest for periprosthetic fracture ($95,364), followed by aseptic loosening ($81,157), PJI ($80,983), RTC rupture ($57,785), implant failure ($55,312), and instability ($52,771, *P* < .001). Ancillary charges were highest for aseptic loosening ($72,505), then periprosthetic fracture ($71,981), PJI ($57,329), RTC rupture ($49,414), implant failure ($46,209), and instability ($42,943, *P* < .001). Accommodation charges were highest for PJI ($23,655) and periprosthetic fracture ($23,383). They were lower for instability ($9829), implant failure ($9104), aseptic loosening ($8652), and RTC rupture ($8370, *P* < .001).

**Table IV:** Revision TSA subgroup analysis of effect of indication on charges.

	Aseptic loosening	Implant failure	Instability	Periprosthetic fracture	Periprosthetic joint infection	RTC rupture	*P* value∗
	*n = 359*	*n = 1024*	*n = 627*	*n = 103*	*n = 640*	*n = 240*	
Accommodation total charges	8652 ± 9758	9104 ± 8858	9829 ± 12,927	23,383 ± 74,063	23,655 ± 27,378	8370 ± 9943	**<.001**
Noncovered accommodation charges	15.58 ± 122.91	76.12 ± 653.77	81.16 ± 667.22	22.62 ± 161.31	167.07 ± 2381.51	19.98 ± 167.25	.5
Ancillary total charges	72,505 ± 53,126	46,209 ± 28,367	42,943 ± 36,128	71,981 ± 65,968	57,329 ± 39,394	49,414 ± 27,600	**<.001**
Noncovered ancillary charges	0.00 ± 0.05	190.37 ± 3716.15	123.50 ± 3082.65	0.05 ± 0.41	17.49 ± 442.22	175.22 ± 2581.53	**.049**
Total charges	81,157 ± 55,874	55,312 ± 31,636	52,771 ± 43,858	95,364 ± 124,776	80,983 ± 54,963	57,785 ± 32,972	**<.001**
Total noncovered charges	15.58 ± 122.91	266.49 ± 4113.12	204.66 ± 3423.37	22.67 ± 161.30	184.56 ± 2810.05	195.19 ± 2585.59	.08

*TSA*, total shoulder arthroplasty; *RTC*, rotator cuff.

Bolded *P*-values denote a statistically significant value. All charges listed in US Dollars.

∗ Kruskal-Wallis rank sum test.

Subgroup analysis was performed to examine the effect of hospital volume on inpatient charges (Table V). For the aTSA group, high-volume centers were able to achieve significantly lower total ($48,478 vs. $53,020, *P* < .001) and ancillary ($41,427 vs. $46,743, *P* < .001) charges compared to low-volume centers. Conversely, high-volume centers had significantly higher accommodation ($7015 vs. $6277, *P* < .001) and noncovered accommodation ($79 vs. $34, *P* < .001) charges than low-volume centers. For rTSA, high-volume centers had lower total ($65,171 vs. $68,022, *P* < .001) and ancillary ($56,854 vs. $59,600, *P* < .001) charges compared to low-volume centers. For HA, high-volume centers had lower total ($50,310 vs. $57,953, *P* < .001), total noncovered ($205 vs. $520, *P* = .04), ancillary ($37,568 vs. $42,677, *P* < .001), noncovered ancillary ($78 vs. $303, *P* = .021), and accommodation charges ($12,743 vs. $15,276, *P* = .005) than those of low-volume centers. For revTSA, high-volume centers had lower total accommodation charges than low-volume centers ($11,192 vs. $14,704, *P* < .001).

**Table V:** Subgroup analysis of effect of hospital volume on inpatient charges.

Anatomic TSA	High	Low	*P* value∗
	*n = 11,722*	*n = 6931*	
Accommodation total charges	7051 ± 6442	6277 ± 7796	**<.001**
Noncovered accommodation charges	78.98 ± 503.63	34.04 ± 664.67	**<.001**
Ancillary total charges	41,427 ± 26,591	46,743 ± 30,174	**<.001**
Noncovered ancillary charges	70.68 ± 1688.05	50.31 ± 1821.46	.4
Total charges	48,478 ± 28,894	53,020 ± 33,451	**<.001**
Total noncovered charges	149.66 ± 1948.74	84.35 ± 2419.84	.056

Reverse TSA	High	Low	*P* value∗
	*n = 10,694*	*n = 8559*	
Accommodation total charges	8317 ± 13,881	8422 ± 13,701	.6
Noncovered accommodation charges	51.89 ± 760.87	37.69 ± 594.23	.15
Ancillary total charges	56,854 ± 40,223	59,600 ± 40,006	**<.001**
Noncovered ancillary charges	43.85 ± 1484.95	68.43 ± 2226.11	.4
Total charges	65,171 ± 45,964	68,022 ± 46,976	**<.001**
Total noncovered charges	95.73 ± 1836.62	106.12 ± 2637.13	.8

Hemiarthroplasty	High	Low	*P* value∗
	*n = 1381*	*n = 2621*	
Accommodation total charges	12,743 ± 25,438	15,276 ± 30,207	**.005**
Noncovered accommodation charges	127.03 ± 1080.77	216.34 ± 3194.22	.2
Ancillary total charges	37,568 ± 28,406	42,677 ± 41,949	**<.001**
Noncovered ancillary charges	78.36 ± 1730.33	303.25 ± 4397.11	**.021**
Total charges	50,310 ± 48,011	57,953 ± 65,122	**<.001**
Total noncovered charges	205 ± 2609	520 ± 6947	**.04**

Revision TSA	High	Low	*P* value∗
	*n = 2673*	*n = 1463*	
Accommodation total charges	11,192 ± 14,629	14,703 ± 30,710	**<.001**
Noncovered accommodation charges	94.41 ± 1254.34	56.44 ± 916.19	.3
Ancillary total charges	52,335 ± 38,838	52,327 ± 40,144	>.9
Noncovered ancillary charges	48.92 ± 1695.05	156.25 ± 3219.90	.2
Total charges	63,527 ± 44,874	67,030 ± 61,591	.056
Total noncovered charges	143.33 ± 2311.66	212.69 ± 3790.31	.5

*TSA*, total shoulder arthroplasty.

Bolded *P*-values denote a statistically significant value. All charges listed in US Dollars.

∗ Welch two-sample t-test.

**Figure 1:**
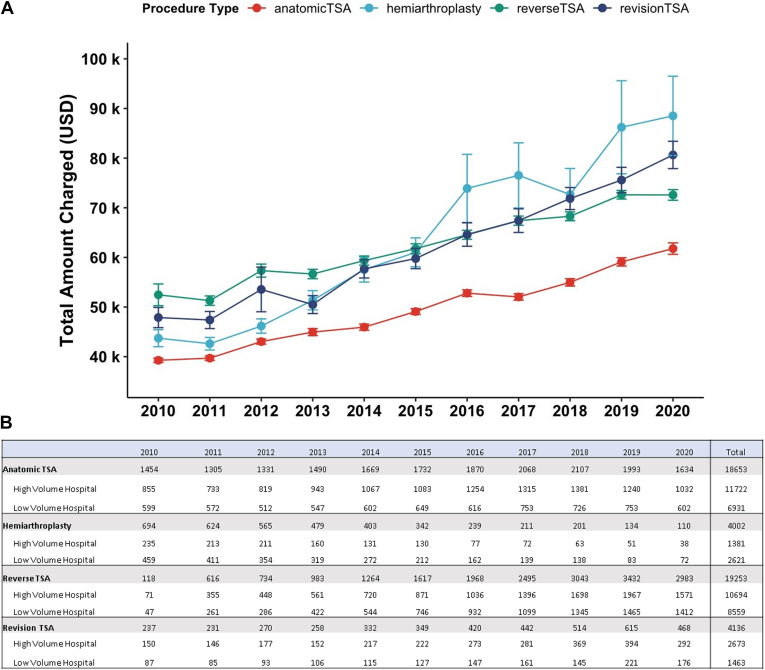
(**A**) Scatter plot of mean inpatient total charges by year for each type of shoulder replacement. Each point represents mean inpatient total charges (USD) for each year +/− standard deviation. Between 2010 and 2020, total charges increased 57.3%, 102.5%, 38.4%, and 68.4% for aTSA, HA, rTSA, and revTSA. Per the US Bureau of Labor Statistics, the rate of inflation during this period was 18.69%. This suggests that hospital charges outpaced inflation by approximately 3, 5.5, 2, and 3.5 times for aTSA, HA, rTSA, and revTSA over the decade. (**B**) Corresponding table depicting the annual incidence of each type of shoulder arthroplasty. *aTSA*, anatomic total shoulder arthroplasty; *HA*, hemiarthroplasty; *rTSA*, reverse total shoulder arthroplasty; *revTSA*, revision total shoulder arthroplasty.

**Figure 2:**
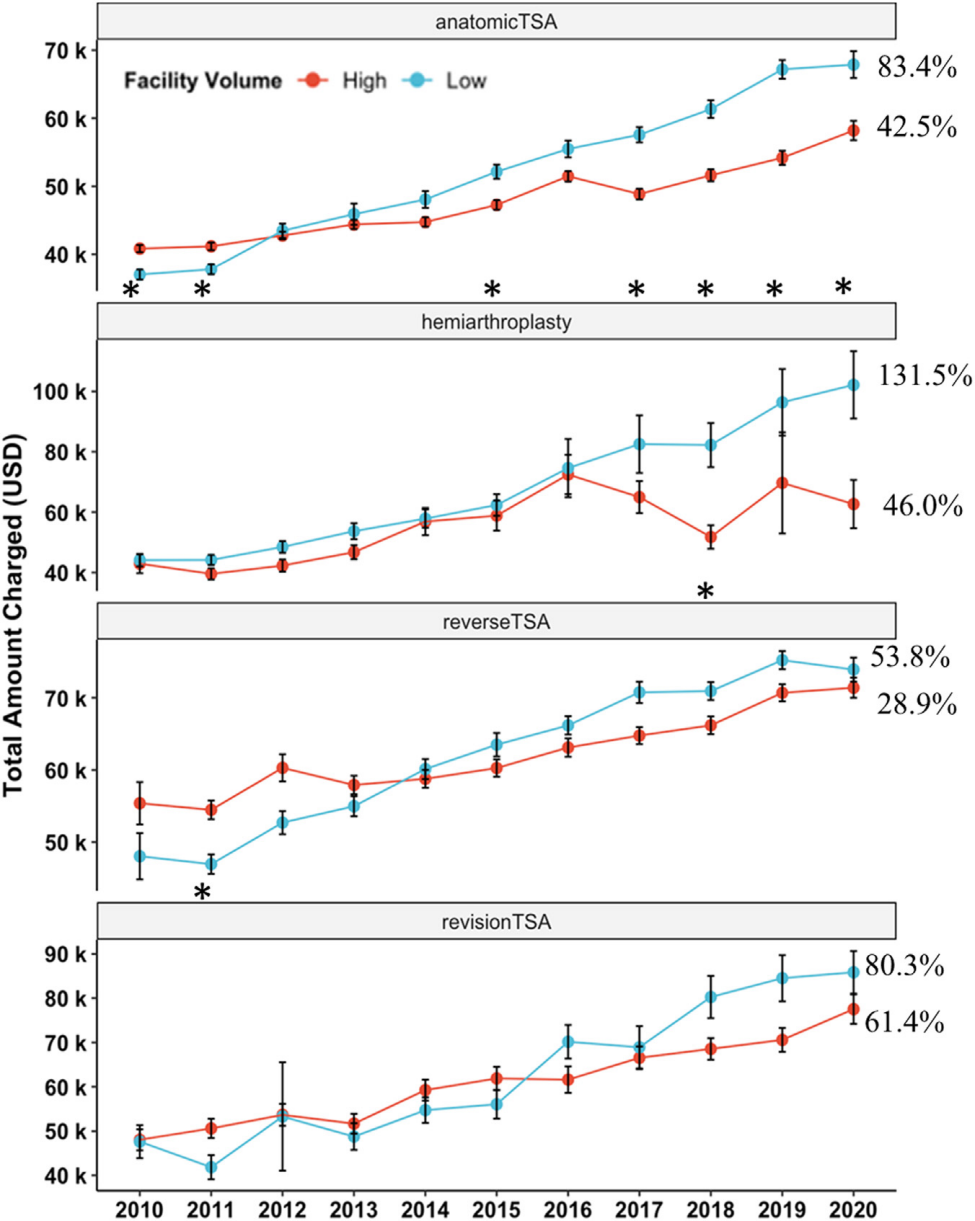
Scatter plot depicting differences in total inpatient charges between high- and low-volume facilities for each type of shoulder arthroplasty. Charge increases over decade are depicted as percent of mean total charge for the 2010 year. *∗* denotes if there is a statistically significant difference (*P* < .05) between high- and low-volume facilities for each calendar year. *TSA*, total shoulder arthroplasty.

Trends in total charges were examined for aTSA, rTSA, HA, and revTSA over the period studied (Fig. 1*A*). From 2010 to 2020, there were increases in total inpatient charges of 57.3%, 102.5%, 38.4%, and 68.4% for aTSA, HA, rTSA, and revTSA. There were changes in the annual incidence of each type of arthroplasty (Fig. 1*B*); the incidence increased 12.4% for aTSA, 97.5% for revTSA, and 2428% for rTSA, and decreased 83.9% for HA. High- and low-volume center subgroups were compared on a per-year basis during the study period (Fig. 2). aTSA total charges increased for both low- and high-volume centers by 83.4% and 42.5%, respectively. In 2010, inpatient charges were significantly higher for patients at high-volume centers (*P* = .001), but by 2020, charges were significantly higher for patients at low-volume centers (*P* = .003). HA total charges increased by 131.5% and 46.0% for low and high-volume centers, although the differences in mean total inpatient charges were only significantly different in the year 2018 (*P* = .011). rTSA total charges for low- and high-volume centers increased by 53.8% and 28.9%, although there were no significant differences between groups for any single year. RevTSA total charge increases for low- and high-volume centers were 80.3% and 61.4%, and there were no significant differences between groups for any single year.

## Discussion

There were significant differences in inpatient charges between HA, aTSA, rTSA, and revTSA. Total charges for all shoulder arthroplasty procedures have increased dramatically during the 2010-2020 decade, outpacing inflation by a factor ranging from 2 to 5.5. rTSA and revTSA had the highest total and ancillary charges, and HA had the highest accommodation charges. Conversely, aTSA had the lowest total charges. High-volume centers achieved significantly lower mean total and ancillary charges for primary arthroplasty and lower rates of total charge increases for all shoulder arthroplasty procedures.

Our primary analysis quantified total inpatient charges to insurers for each of the major types of shoulder arthroplasty. Our findings are generally consistent with prior studies showing that rTSA is associated with both higher total and ancillary inpatient charges compared to other forms of primary shoulder arthroplasty.^3^^,^^8^^,^^21^^,^^22^ These higher total charges likely stem from high ancillary charges, which is expected as implant/supply expenses fall within this category. Prior studies have attributed the relatively higher charges of rTSA to implant and operative expenses.^3^^,^^4^^,^^8^^,^^21^ In our study, HA was found to have the highest accommodation charges and inpatient length of stay. These two findings are likely related, driven by the large portion of patients undergoing HA for fracture. Prior comparisons of HA and rTSA charges found initial inpatient charges for rTSA performed for proximal humerus fractures were significantly higher than those of HA, but total charges at 1-year were similar due to higher postoperative charges related to physical therapy in the HA group.^21^ Nevertheless, inpatient charges may represent the largest component of expenses for shoulder arthroplasty perioperative care and, furthermore, inpatient/operative expenses often have the greatest potential for cost mitigation.^8^^,^^28^

Although rTSA was found to have the highest total charges, revTSA total charges were close behind, as the majority of revisions involve use of rTSA implants. Subgroup analysis found that revision for periprosthetic fracture, followed by aseptic loosening, and PJI were associated with notably higher total inpatient charges than those for implant failure, instability, and RTC rupture. Periprosthetic fracture and aseptic loosening groups had the highest ancillary charges, likely stemming from additional operative expenses. Periprosthetic fractures most often occur on the humerus and may require large revision humeral components and/or plates.^20^ Revision for aseptic loosening commonly necessitates bone grafting, glenoid augments, and/or revision components to reconstruct osteolysis of the glenoid and humerus, all of which are associated with higher operative charges.^8^^,^^9^^,^^12^^,^^20^^,^^30^ Revision for PJI may require expensive reconstructive components, but we were unable to differentiate between the first and second stages of revision within the database, and there exists significant differences in implant charges between an antibiotic-eluting hemispacer and definitive revision components. This limitation may explain why ancillary charges for PJI are below those for periprosthetic fracture and aseptic loosening but above other indications for revTSA. Periprosthetic fracture and PJI groups exhibited high accommodation charges, which are likely driven by utilization of greater amounts of hospital resources in the perioperative period.

There exists limited and conflicting literature regarding the impact of hospital volume on inpatient hospital charges for shoulder arthroplasty. Carducci et al performed a cost analysis of 1571 shoulder arthroplasties at four high-volume institutions, and no significant correlations were found between hospital case volume and inpatient charges for rTSA and aTSA.^4^ In contrast, Gregory et al, using the Texas Health Care Information Collection Database, compared charges between patients undergoing inpatient and outpatient TSA to show that high-volume inpatient TSA incurred lower average charges as compared to low volume inpatient TSA.^24^ Our study found that high hospital volume correlated with lower total and ancillary charges for all types of primary TSA but not for revTSA. High-volume centers had lower accommodation charges for revTSA and HA but higher charges for aTSA. Generally, these findings support those of Gregory et al for most types of shoulder arthroplasty.

The landscape of the healthcare system has greatly shifted with a concentration on reducing health-care expenditure while improving patient care. There remains a great need for the understanding of the relationship between inpatient costs, payments, and hospital charges as TSA utilization continues to rise.^2^^,^^6^^,^^10^^,^^13^ It is interesting to note that total inpatient charges for all types of shoulder arthroplasty outpaced the inflation rate from the years 2010 to 2020. Per the Bureau of Labor Statistics, the inflation rate during this time period was 18.7%, suggesting that hospital charges outpaced inflation by approximately 3, 5.5, 2, and 3.5 times for aTSA, HA, rTSA, and revTSA.^27^ This is consistent with prior data that reported an increase in total hospital charges of 99.9% during the years 2005-2014. We found that high-volume shoulder arthroplasty centers achieved a lower rate of charge increase for all categories of arthroplasty as compared to low-volume centers. This suggests that high-volume centers are more economical compared to low-volume centers, although we did not correlate these charge differences with postoperative complications, readmissions, or revisions. Understanding trends in inpatient charges and the effect of hospital procedure volume may provide valuable insight for hospital administrators and public health officials as they develop cost-mitigating strategies in response to rising health-care expenditures in the United States.

It is interesting to note that despite steady increases in hospital charges for shoulder arthroplasty, physician reimbursement for this group of procedures has either stagnated relative to inflation or fallen over the past decade.^5^^,^^16^^,^^17^ Lopez et al found that from the years 2012 to 2017, after adjusting for inflation, Medicare reimbursement to physicians fell 7.1% and 11.8% for primary and revision TSA.^16^ In another study that reviewed charges and payments during the years 2005-2014, Casp et al found that hospital charges ($33,836-$67,177, +99.9%) and payments ($8758-$14,167, +61.8%) both increased, but physician charges have stagnated ($4284-$4674, +9.1%) and physician payments have decreased ($1028-$884, −14%).^5^ Further research may elucidate this discordance between physician and hospital reimbursement to better inform physician advocacy groups.

There are several notable limitations to this study. Our study compares inpatient charges, and one must be aware that costs, payments, and charges are not synonymous; there exists a complex negotiating process between providers and payers that ultimately determines payments. Hospitals often only receive compensation for a fraction of requested charges, and therefore, the reported charges overestimate actual payments. Payments to hospitals vary widely depending on a variety of factors (patient demographics, insurance type, level of care, etc.), nevertheless inpatient payments can be estimated with moderate accuracy for most hospital systems using predictive modeling to determine mean payment-to-charge ratios (PCR). PCR values range from 0 to 1 and are determined by dividing the amount paid to a hospital by the amount charged by a hospital. Smith et al found that PCRs ranged from 0.320 to 0.630 depending on insurance type, whereas Medicare reimbursed a PCR of 0.320, private insurance reimbursed a PCR of 0.487, and other payers (workers compensation programs, etc.) reimbursed a PCR of 0.611.^26^ Hospital inpatient costs also have great variability but can be estimated using a hospital charge-to-cost ratio. Although charge-to-cost ratios generally correlate with PCRs, the two ratios are not synonymous. We acknowledge that our analysis is limited only to hospital charges, but it remains important for orthopedists and healthcare policymakers to understand the trends in inpatient charges as charges, payments, and costs are interconnected.

Other limitations are that the SPARCS database is representative of New York State, and the charges for shoulder arthroplasty may exhibit slight regional differences. SPARCS is also an administrative database, and our analysis was limited by the variables available and the data granularity. As with all administrative database studies, when considering tens of thousands of patients, it isn’t feasible to manually confirm the primary diagnosis associated with each encounter, which may limit our ability to accurately discern between specific surgical indications. This analysis is also susceptible to diagnosis coding errors. This is particularly evident when trying to differentiate between PJI and aseptic loosening for revision TSA, as intraoperative culture data was not available and some cases reported as aseptic loosening may represent PJI. Furthermore, we did not consider socioeconomic or census data in the analysis and these factors are likely to play a role in inpatient charges. Socioeconomic limitations are somewhat mitigated by the large and diverse patient population analyzed. Furthermore, we focused on inpatient charges during the single encounter and did not consider postoperative outcomes, complications, and charges associated with postdischarge care. Because of this, we were unable to determine if hospital charges were at all correlated with outcomes.

## Conclusion

Total inpatient charges for all arthroplasty types increased dramatically from 2010 to 2020, outpacing inflation rates, but high-volume centers demonstrated greater success at mitigating charge increases as compared to low-volume centers. Total charges were the highest for rTSA and revTSA and the lowest for aTSA.

## Disclaimers

Funding: No outside funding or grants were received in support of completion of this study.

Conflict of interest: The authors, their immediate families, and any research foundation with which they are affiliated have not received any financial payments or other benefits from any commercial entity related to the subject of this article.
